# Optical Quality Assessment in Patients with Macular Diseases Using Optical Quality Analysis System

**DOI:** 10.3390/jcm8060892

**Published:** 2019-06-21

**Authors:** Joon Hee Cho, So Hyun Bae, Ha Kyoung Kim, Young Joo Shin

**Affiliations:** 1 Department of Ophthalmology, Kangnam Sacred Heart Hospital, Hallym University College of Medicine, Seoul 07441, Korea; iamjooni@gmail.com (J.H.C.); vitric79@naver.com (S.H.B.); 5193eye@naver.com (H.K.K.); 2 Hyemin Eye Hospital, Seoul 05829, Korea

**Keywords:** macular diseases, optical quality analysis system, objective scattering index, modulation transfer function

## Abstract

Macular diseases cause vision loss, as the macula is the functional center for vision. In this study we assessed optical quality in eyes with macular diseases and evaluated the effectiveness of the Optical Quality Analysis System (OQAS) to detect macular diseases. We analyzed 88 eyes of 88 patients with macular diseases (51 men and 37 women; mean age: 65.24 ± 12.96 years) and 43 eyes of 43 control subjects (11 men and 32 women; mean age: 54.70 ± 15.03 years). The macular diseases included age-related macular disease (*n* = 62), diabetic macular edema (*n* = 19), and retinal vein occlusion (*n* = 7). We measured the objective scattering index (OSI), modulation transfer function (MTF), Strehl ratio, and predicted visual acuities (PVAs) at 100, 20, and 10% contrast levels in both groups using OQAS. We measured the retinal thickness in the macular disease group on optical coherence tomography. The macular disease and control groups significantly differed in OSI, MTF, Strehl ratio, and PVAs at 20 and 10% contrast levels (*p* < 0.05). In the macular disease group, retinal thickness correlated with OSI (r = 0.370, *p* < 0.001) and MTF (r = −0.264, *p* = 0.014). In conclusion, optical quality was worse in the patients with macular diseases compared to the control subjects, and retinal thickness affected optical quality. Therefore, the distortion of macular shape may contribute to vision disturbance in patients with macular diseases.

## 1. Introduction

Macular diseases cause vision loss, as the macula is the functional center for vision [1]. Age-related macular degeneration (AMD) and macular edema are the most common macular diseases [2]. Early detection of macular diseases is important because early treatment can preserve vision [2]. Various methods, including optical coherence tomography (OCT) and fluorescein angiography, have been used to detect macular diseases [3]. However, fluorescein angiography has the drawback of intravenous fluorescein injections, and neither method qualitatively assesses functions of the macula [3]. Changes in retinal morphology and disruption of photoreceptors in the retina can lead to poor visual acuity.

The Optical Quality Analysis System (OQAS) was developed to measure visual quality using the objective scattering index (OSI), modulation transfer function (MTF), Strehl ratio, and predicted visual acuities (PVAs) at contrast levels of 100, 20, and 10% [4]. OQAS calculates the point spread function by detecting laser light reflected from the retinal surface through a double-pass system [5,6]. Therefore, it has mostly been used to evaluate lens opacity [6], the effect of intraocular lenses on vision quality [7], and corneal surface disorders [8,9]. However, as laser light passes through the ocular media and is reflected from the retinal surface [5], OQAS could probably be used to detect retinal surface distortions. A previous OQAS-based study revealed that macular edema due to central serous chorioretinopathy affects optical quality and ocular scattering [10], but no study has yet used OQAS to assess macular edema secondary to AMD, diabetic retinopathy (DR), or retinal vein occlusion (RVO). 

In this study, we assessed optical quality in eyes with macular diseases using OQAS. 

## 2. Methods

This study was approved by the Hallym University Kangnam Sacred Heart Hospital’s Institutional Review Board and conducted in accordance with the principles of the Declaration of Helsinki. The medical records of patients who underwent assessment using OQAS (Visiometrics, Terrassa, Spain) at Kangnam Sacred Heart Hospital from December 2014 to May 2015 were evaluated. Because of the study’s retrospective nature, the Institutional Review Board waived the requirement for participant consent.

To eliminate the influence of anterior segment abnormalities, we excluded patients with a cataract more advanced than grade 1, corneal abnormalities, or vitreous opacity. Patients with monofocal pseudophakia were included because it does not impair optical quality [11]. The macular disease group consisted of patients with macular thicknesses >300 μm because of AMD, DR, or RVO who needed intravitreal anti-vascular endothelial growth factor (anti-VEGF) injections. These patients underwent fundus examination, macular OCT (Cirrus; Carl Zeiss Meditec, Jena, Germany), and OQAS examination before the anti-VEGF injections. The control group consisted of patients with mild dry eye syndrome, without ocular surface abnormality, who had undergone OQAS examination. These patients had no retinal or corneal abnormalities and no ocular abnormalities other than mild ocular discomfort. 

OQAS was used to measure OSI, MTF, Strehl ratio, and PVAs at 100, 20, and 10% contrast in both groups. PVA was expressed as the logarithm of the minimum angle of resolution (logMAR). In the macular disease group, the retinal thickness was measured on sectional OCT based on the Early Treatment of Diabetic Retinopathy Study. The central subfield thickness and cube average thickness were measured.

### Statistics

Data are presented as mean ± standard deviation. All data obtained were used for statistical analysis. Independent *t*-tests were used to compare the macular disease and control groups. The normality of data samples was checked using the Shapiro-Wilk test. The Pearson coefficient was calculated to correlate between retinal thickness and optical quality. One-way analysis of variance (ANOVA) with Bonferroni post-hoc correction was used to compare optical quality among the AMD, DR, and RVO subgroups. Statistical analyses were conducted using the Statistical Package for the Social Sciences version 24.0 for Windows (IBM, Armonk, NY, USA), and statistical significance was defined as *p* < 0.05. 

## 3. Results

The macular disease group included 88 eyes of 88 patients with macular diseases, and the control group included 43 eyes of 43 patients. Table 1 shows the baseline characteristics and optical quality of both groups. The mean age of the macular disease group was significantly greater than that of the control group (*p* < 0.001). The male-to-female ratio was 11:32 (25.6%; 74.4%) in the control group and 51:37 (58.0%; 42.0%) in the macular disease group. The mean visual acuity was 0.06 ± 0.19 logMAR units in the control group and 0.44 ± 0.33 logMAR units in the macular disease group (*p* < 0.001). Pseudophakia was present in four (9.30%) patients in the control group and in 36 (40.91%) patients in the macular disease group. In the macular disease group, the mean central subfield thickness and cube average thickness were 329.4 ± 113.3 and 353.4 ± 81.1 μm, respectively. 

**Figure 1:**
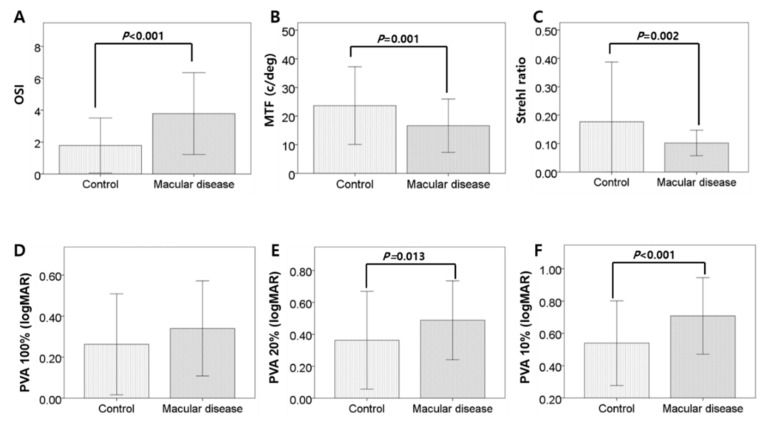
Comparison of parameters of optical quality in both groups. **A, B, C**. The group of macular disease had significantly higher mean OSI, lower mean MTF, and lower mean Strehl ratio. **D, E, F**. Predicted visual acuities (PVA) at 10% and 20% contrast levels showed significantly higher in the macular disease, but no significant difference at 100% contrast. P-value is presented if it isstatistically significant by independent *t*-test. Error bars represent standard deviation (SD).

Compared to the control group, the macular disease group had significantly higher mean OSI (1.78 ± 1.73 vs. 3.78 ± 2.56; *p* < 0.001, independent *t*-test), lower mean MTF (23.64 ± 13.56 vs. 16.66 ± 9.32; *p* = 0.001), and lower mean Strehl ratio (0.18 ± 0.21 vs. 0.10 ± 0.45; *p* = 0.002). The macular disease group also had significantly higher mean PVAs at 10% (0.54 ± 0.26 vs. 0.71 ± 0.24; *p* < 0.001) and 20% contrast levels (0.36 ± 0.31 vs. 0.49 ± 0.25; *p* = 0.013) but no significant difference at 100% contrast (0.26 ± 0.24 vs. 0.34 ± 0.23; *p* = 0.081) (Figure 1). These differences indicate lower optical quality in the macular disease group. 

**Figure 2:**
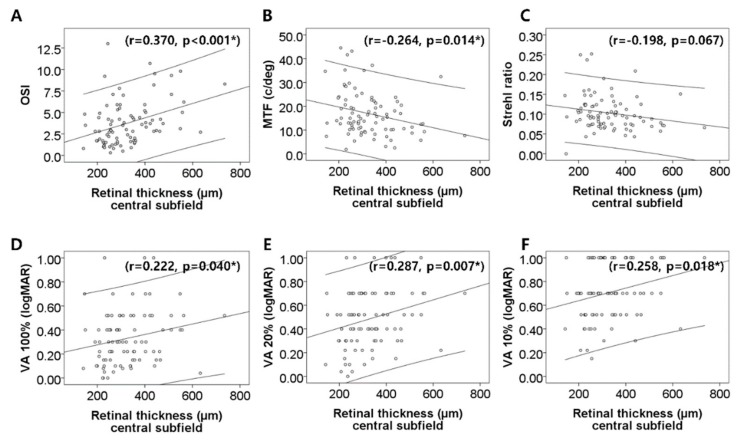
Correlation between central subfield retinal thickness and six optical quality values. **A, B, C**. Positive correlation is showed in OSI, and negative correlation is showed in MTF, Strehl ratio. **D, E, F**. Also, the central macular thickness positively correlated with predicted visual acuities(PVA) at contrast levels of 100%, 20%, and 10%. * Statistically significant by Pearson correlation test.

**Figure 3:**
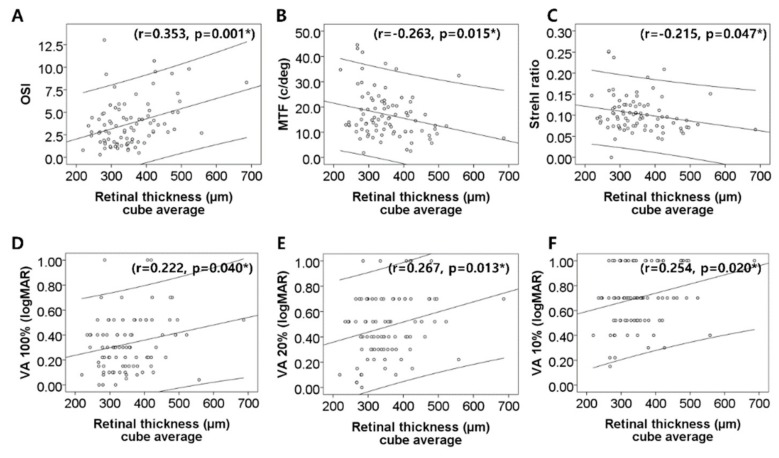
Correlation between the retinal thickness of cube average and optical quality value. **A, D, E**, **F**. Positive correlation with retinal thickness is showed in OSI and predicted visual acuities (PVA) at contrast levels of 100%, 20%, and 10%. **B, C**. While MTF and Strehl ratio showed negative correlation with retinal thickness. * Significant by Pearson correlation test.

In the macular disease group, central subfield thickness positively correlated with OSI (r = 0.370, *p* < 0.001, Pearson’s correlation test) and PVAs at contrast levels of 100% (r = 0.222, *p* = 0.040), 20% (r = 0.287, *p* = 0.007), and 10% (r = 0.258, *p* = 0.018), while it negatively correlated with MTF (r = −0.264, *p* = 0.014) (Figure 2). The cube average thickness positively correlated with OSI (r = 0.353, *p* = 0.001) and PVAs at contrast levels of 100% (r = 0.222, *p* = 0.040), 20% (r = 0.267, *p* = 0.013), and 10% (r = 0.254, *p* = 0.020), while it negatively correlated with MTF (r = −0.263, *p* = 0.015) and Strehl ratio (r = −0.215, *p* = 0.047) (Figure 3). 

**Figure 4:**
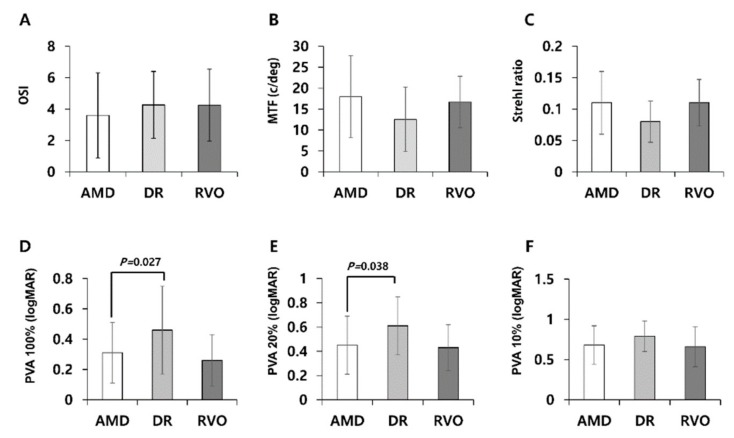
Comparison among subgroups of age-related macular degeneration (AMD), diabetic retinopathy with macular edema (DR), and retinal vein occlusion with macular edema (RVO). **D, E**. Compared to the AMD subgroup, the DR subgroup had higher mean predicted visual acuities (PVA) at 100% and 20% contrast levels. **A, B, C, F**. OSI, MTF, Strehl ratio, or PVA at 10% contrast did not showed significant inter-subgroup differences. P-value is presented if it is significant by Bonferroni post hoc test. Error bars represent standard deviation (SD).

In the macular disease group, 66, 19, and seven patients had AMD, DR, and RVO, respectively (Table 2, Figure 4). These subgroups did not significantly differ in logMAR visual acuities, OSI, MTF, Strehl ratio, or PVAs at 10% contrast. However, ANOVA revealed significant inter-subgroup differences in PVAs at contrast levels of 100% (*p* = 0.023) and 20% (*p* = 0.036). Compared to the AMD subgroup, the DR subgroup had higher mean PVAs at 100% (0.31 ± 0.20 vs. 0.46 ± 0.29; *p* = 0.027, Bonferroni post hoc test) and 20% contrast levels (0.45 ± 0.24 vs. 0.61 ± 0.24; *p* = 0.038). These results indicate lower optical quality in eyes with DR. 

## 4. Discussion

We aimed to determine whether OQAS can distinguish eyes with macular diseases from control eyes and found that the OQAS optical quality parameters, including OSI, MTF, and Strehl ratio, were worse in the macular disease group compared to the control group. 

OSI, which represents intraocular scattered light, is the ratio of the light in the peripheral zone to that in the central zone of the retinal image [12], and MTF and Strehl ratio are calculated from the frequency of reflected light [12]. These facts suggest several mechanisms that may explain our observations. First, macular diseases are accompanied with edematous and irregular retinal surfaces [1], which may affect the reflected intraocular light and worsen optical quality. This is consistent with the correlation observed between retinal thickness and optical quality (Figure 2 and Figure 3). Although the correlation coefficients were low, the correlation was statistically significant. Low correlation coefficients may be because of the small sample size [13]. Second, retinal changes at the photoreceptor inner segment/outer segment (IS/OS) junction influence optical quality and intraocular light scattering [10]. In eyes with diabetic macular edema, decreased retinal sensitivity correlates with IS/OS junction disruption [14]. The photoreceptor outer segment is thinner in patients with diabetic macular edema compared to the controls or patients with DR without macular edema [15]. In eyes with macular edema secondary to RVO, the photoreceptor layer thickness is associated with visual function [16]. Furthermore, photoreceptor loss occurs in AMD [17]. In our study, all eyes in the macular disease group had macular edema, so photoreceptor disruption might have contributed to the worsening of OQAS parameters. Furthermore, deterioration of optical quality because of retinal surface distortion and photoreceptor abnormalities within the macula may contribute to visual disturbances. 

Our analysis of the macular disease subgroups revealed no significant differences in OQAS parameters, except for PVAs at 100 and 20% contrast levels, which were significantly worse in the DR subgroup than in the AMD subgroup. This difference may have arisen from differences in retinal thickness. Subfoveal and cube average thicknesses were greater in the DR subgroup than in the AMD subgroup, and greater retinal thickness is associated with higher retinal contrast sensitivity [18]. 

This study has several limitations. First, the macular disease and control groups differed in age. However, we excluded individuals with major age-related conditions that disrupt optical quality, such as cataract, vitreous opacity, and corneal diseases. We did not exclude individuals with monofocal pseudophakia because it does not impair optical quality [11], and the macular disease group had more pseudophakic eyes compared to the control group. Second, our control group consisted of patients with mild dry eye syndrome. However, they had no ocular abnormalities other than ocular discomfort and tear breakup time of <10 s, and these conditions only minimally impaired optical quality. In this study, OCT was not performed in the control group because the controls had no macular or retinal abnormalities. The retinal thickness was not compared between the control and macular disease groups.

In conclusion, optical quality was lower in the macular disease group than in the control group. Deterioration of optical quality because of retinal thickness distortions and photoreceptor abnormalities in the macula may contribute to visual disturbances in patients with macular diseases. OQAS was effective in detecting eyes with macular diseases, with OSI being the most useful parameter. However, OQAS was not effective in diagnosing the specific macular disease. OQAS may be useful in detecting visual disturbance due to macular diseases. 

## Figures and Tables

**Table 1:** Baseline characteristics and comparison of optical quality values in patients with macular disease and patients in the control group.

Characteristics	Control	Macular Disease	*p*-Value
**Eyes (*n*)**	43	88	
**Age (years)**	54.70 ± 15.03	65.24 ± 12.96	<0.001 *
**Male:female**	11:32	51:37	
**Visual acuity (logMAR)**	0.06 ± 0.19	0.44 ± 0.33	<0.001 *
**No. of pseudophakic eyes (*n*, %)**	4 (9.30%)	36 (40.91%)	
**Retinal thickness**			
**Central subfield (µm)**		329.4 ± 113.3	
**Cube average (µm)**		353.4 ± 81.1	
**Optical quality**			
**OSI (OSI value)**	1.78 ± 1.73	3.78 ± 2.56	<0.001 *
**MTF cut-off (c/deg)**	23.64 ± 13.56	16.66 ± 9.32	0.001 *
**Strehl ratio**	0.18 ± 0.02	0.10 ± 0.04	0.002 *
**PVA at 100% (logMAR)**	0.26 ± 0.24	0.34 ± 0.23	0.081
**PVA at 20% (logMAR)**	0.36 ± 0.31	0.49 ± 0.25	0.013 *
**PVA at 10% (logMAR)**	0.54 ± 0.26	0.71 ± 0.24	<0.001 *

OSI; optical scattering index, MTF; modulation transfer function, PVA; predicted visual acuity. Data are expressed as mean ± SD. * Statistically significant by independent *t*-test.

**Table 2:** Comparison of subgroups of three different diseases including age macular degeneration, diabetic retinopathy, and retinal vein occlusion.

Characteristics	Subgroup of Macular Disease			*p*-Value
	AMD	DR	RVO	
**Eyes (*n*)**	62	19	7	
**Age (years**)	68.35 ± 11.28	56.42 ± 14.01	61.57 ± 13.76	0.001 *
**Male:female**	40:22	9:10	2:5	
**Visual acuity (logMAR)**	0.44 ± 0.36	0.49 ± 0.23	0.31 ± 0.22	0.453
**Retina thickness**				
**Subfovea (µm)**	284.1 ± 81.3	421.3 ± 91.2	488.0 ± 128.2	<0.001 *
**Cube average (µm)**	318.9 ± 53.8	427.0 ± 60.7	464.1 ± 105.7	<0.001 *
**Optical quality**				
**OSI**	3.59 ± 2.71	4.26 ± 2.13	4.25 ± 2.29	0.534
**MTF cut-off (c/deg)**	17.93 ± 9.78	12.52 ± 7.69	16.68 ± 6.16	0.085
**Strehl ratio**	0.11 ± 0.05	0.08 ± 0.03	0.11 ± 0.04	0.050
**PVA at 100% (logMAR)**	0.31 ± 0.20	0.46 ± 0.29	0.26 ± 0.17	0.023 *
**PVA at 20% (logMAR)**	0.45 ± 0.24	0.61 ± 0.24	0.43 ± 0.19	0.036 *
**PVA at 10% (logMAR)**	0.68 ± 0.24	0.79 ± 0.19	0.66 ± 0.25	0.232

AMD; age-related macular degeneration, DR; diabetic retinopathy, RVO; retinal vein occlusion; OSI; optical scattering index, MTF; modulation transfer function, PVA; predicted visual acuity; * statistically significant by ANOVA.

## Abbreviations

OQAS	Optical Quality Analysis System
AMD	age-related macular degeneration
OCT	optical coherence tomography
OSI	objective scattering index
MTF	modulation transfer function
PVAs	predicted visual acuities
DR	diabetic retinopathy
RVO	retinal vein occlusion
VEGF	vascular endothelial growth factor
logMAR	logarithm of the minimum angle of resolution
ANOVA	analysis of variance
IS/OS	inner segment/outer segment
